# Pharmacological inhibition of asparaginyl endopeptidase by δ-secretase inhibitor 11 mitigates Alzheimer’s disease-related pathologies in a senescence-accelerated mouse model

**DOI:** 10.1186/s40035-021-00235-4

**Published:** 2021-03-31

**Authors:** Ju Wang, Hui-Jie Hu, Zi-Kai Liu, Jing-Jing Liu, Shan-Shan Wang, Qing Cheng, Hong-Zhuan Chen, Mingke Song

**Affiliations:** 1 Department of Pharmacology and Chemical Biology, Institute of Medical Sciences, Shanghai Jiao Tong University School of Medicine, Shanghai, 200025 China; 2 Institute of Interdisciplinary Integrative Biomedical Research, Shanghai University of Traditional Chinese Medicine, Shanghai, 201210 China

**Keywords:** Alzheimer’s disease, Asparaginyl endopeptidase, Legumain, SAMP8 mouse, δ-Secretase inhibitor 11, Therapeutic target

## Abstract

**Background:**

Currently, there is no cure for Alzheimer’s disease (AD). Therapeutics that can modify the early stage of AD are urgently needed. Recent studies have shown that the pathogenesis of AD is closely regulated by an endo/lysosomal asparaginyl endopeptidase (AEP). Inhibition of AEP has been reported to prevent neural degeneration in transgenic mouse models of AD. However, more than 90% of AD cases are age-related sporadic AD rather than hereditary AD. The therapeutic efficacy of AEP inhibition in ageing-associated sporadic AD remains unknown.

**Methods:**

The senescence-accelerated mouse prone 8 (SAMP8) was chosen as an approximate model of sporadic AD and treated with a selective AEP inhibitor,: δ-secretase inhibitor 11. Activation of AEP was determined by enzymatic activity assay. Concentration of soluble amyloid β (Aβ) in the brain was determined by ELISA. Morris water maze test was performed to assess the learning and memory-related cognitive ability. Pathological changes in the brain were explored by morphological and western blot analyses.

**Results:**

The enzymatic activity of AEP in the SAMP8 mouse brain was significantly higher than that in the age-matched SAMR1 mice. The half maximal inhibitory concentration (IC_50_) for δ-secretase inhibitor 11 to inhibit AEP *in vitro* is was around 150 nM. Chronic treatment with δ-secretase inhibitor 11 markedly decreased the brain AEP activity, reduced the generation of Aβ_1–40/42_ and ameliorated memory loss. The inhibition of AEP with this reagent not only reduced the AEP-cleaved tau fragments and tau hyperphosphorylation, but also attenuated neuroinflammation in the form of microglial activation. Moreover, treatment with δ-secretase inhibitor 11 prevented the synaptic loss and alleviated dendritic disruption in SAMP8 mouse brain.

**Conclusions:**

Pharmacological inhibition of AEP can intervene and prevent AD-like pathological progress in the model of sporadic AD. The up-regulated AEP in the brain could be a promising target for early treatment of AD. The δ-secretase inhibitor 11 can be used as a lead compound for translational development of AD treatment.

**Supplementary Information:**

The online version contains supplementary material available at 10.1186/s40035-021-00235-4.

## Background

Alzheimer’s disease (AD) is the most common form of dementia in the elderly, featured by amyloid β-proteins (Aβ) accumulation and tau aggregation. Currently, there is no cure for AD [1]. Symptomatic therapeutics such as cholinesterase inhibitors and memantine cannot stop AD progression [2, 3]. To achieve therapeutic success, it is essential to apply interventions towards preclinical or early stage of AD [4, 5, 6]. Age is known as the greatest risk factor for AD, and the early events that drive deposition of Aβ and tau during aging have been reported by researchers at Emory University [7, 8, 9]. By analyzing brain tissues of AD patients and mouse models, they have found that the endo/lysosomal asparaginyl endopeptidase (AEP) or legumain is elevated and activated during ageing [10, 11], and that the activation of AEP is a crucial step that links ageing to tau cleavage and processing of amyloid precursor protein (APP) at the early stage of AD [10, 12]. AEP acts as a δ-secretase that cleaves APP and microtubule-associated protein tau, thus playing a pivotal role in the pathogenesis of AD [11, 13]. Interventions targeting AEP may offer potential therapies in the early stage of disease [14].

Previously developed AEP inhibitors are mainly peptidyl compounds including aza-asparaginyl Michael acceptors, aza-peptidyl Asn epoxides and those based on the aza-asparaginyl scaffold [11]. These inhibitors cannot penetrate the blood-brain barrier (BBB). Most recently, a potent and specific AEP/δ-secretase inhibitor 7-morpholin-4-yl-benzo[1,2,5]oxadiazol-4-ylamine (PubChem CID: 1095027) has been identified by the high-throughput screening method [14]. This new AEP inhibitor was named compound 11, and now is called δ-secretase inhibitor 11. It can cross the BBB of mice after oral administration, without induction of long-term systemic toxicity [14]. As expected, the δ-secretase inhibitor 11 can suppress brain AEP activity, reduce tau cleavage, prevent neural degeneration and alleviate memory loss in tau P301S transgenic mice [14], which are generated by introducing mutations of the familial AD (FAD) [15]. However, patients with FAD account for only 5% of AD cases. More than 90% of AD cases are sporadic AD (SAD) and the incidence of SAD increases closely with ageing [16]. The therapeutic efficacy of AEP inhibition remains unknown in the context of age-related SAD. The potential of AEP inhibition in SAD needs to be verified at least in an ageing-associated AD model.

In this study, we assessed the effect of chronic AEP inhibition by δ-secretase inhibitor 11 on AD-like pathological changes in senescence-accelerated mouse prone 8 mice (SAMP8) mice. SAMP8 is an accelerated aging model which has an AD-related pathology including tau hyperphosphorylation, inflammatory response and cognitive impairment observed in AD patients [17, 18]. This strain is gradually being accepted as a relevant model for AD or dementia, and used for testing several preventative or therapeutic interventions [19, 20]. The senescence-accelerated mouse–resistant 1 (SAMR1) mice were used as a control strain for SAMP8 [18].

## Materials and methods

### Animals and ethical statement

Animal experimental procedures were approved by the Animal Experimentation Ethics Committee and Institutional Animal Care and Use Committee (IACUC) at Shanghai Jiao Tong University School of Medicine, and carried out strictly in accordance with the guidelines of Association for Assessment and Accreditation of Laboratory Animal Care [21]. Male SAMP8 and SAMR1 mice were provided by the Beijing Vital River Laboratory Animal Science and Technology Co., Ltd., China. Animals were housed in the SPF animal facility at 24 ± 2 °C under a 12 h/12 h light/dark cycle with free access to water and a standard rodent diet. Four-month-old SAMP8 mice were chosen for experiments and were randomly divided into two groups (*n* = 15/group): δ-secretase inhibitor 11 treatment and vehicle treatment groups. The SAMP8 mice were treated with δ-secretase inhibitor 11 (10 mg/kg) or vehicle once daily *via* oral gavage over a period of 3 months.

### Reagents and antibodies

The AEP inhibitor δ-secretase inhibitor 11 (7-morpholin-4-yl-benzo[1,2,5]oxadiazol-4-ylamine, PubChem CID: 1095027) was purchased from J&K Scientific Ltd. (Beijing, China). The δ-secretase inhibitor 11 was first dissolved in dimethyl sulfoxide as the stock solution and then diluted in 0.9% NaCl solution containing gum arabic for systemic treatment. The primary antibody for mature AEP was from R&D Systems Inc. (Minneapolis, MN). Antibodies for β-actin, Iba1, and microtubule-associated protein 2 (MAP-2) were purchased from Abcam (Cambridge, MA). Antibody for AEP-cleaved tau (N368) was from Sigma-Aldrich (St. Louis, MO). Antibody for phospho-tau (Thr231) was from Thermo Fisher Scientific (Carlsbad, CA). Antibodies for postsynaptic density protein 95 (PSD-95) and synaptophysin (SYP) were from Cell Signaling Technology (Danvers, MA).

### Enzymatic activity assay and ELISA

Recombinant mouse AEP (R&D Systems Inc., Minneapolis, MN) was diluted to 50 μg/ml in activation buffer (0.1 M NaOAc, 0.1 M NaCl, pH 4.5), incubated for 6 h at 37 °C with or without AEP inhibitor, and then diluted to 2 ng/μl in assay buffer (50 mM MES, 250 mM NaCl, pH 5.5). Fifty microliter of 2 ng/μlL AEP was loaded in a plate, and added with 50 μl of 200 μM substrate Z-Ala-Ala-Asn-AMC (Bachem AG, Switzerland) diluted with assay buffer. Absorbance was read at excitation and emission wavelengths of 380 nm and 460 nm (top read), respectively, in kinetic mode for 45 min. Tissue homogenate (10 μg) was incubated in 200 μl of assay buffer containing 20 μM AEP substrate and assayed as above. The activity of AEP was expressed as the reading at 45 min minus the first reading. The levels of soluble Aβ_1–40_ and Aβ_1–42_ in the whole brain homogenate were determined using the ELISA kit (BlueGene Biotech, Shanghai, China) according to the manufacturer’s recommendations.

### Western blot analysis

Mice were deeply anesthetized with 4% isoflurane (RWD Life Science, Shenzhen, China) and then decapitated. Brain tissues were collected and lysed in RIPA buffer (Beyotime, Nanjing, China) supplemented with protease inhibitor (Beyotime). The total protein concentration was determined using the BCA Protein Assay Kit (Sangon Biotech, Shanghai, China). Equal amounts of protein extracts (30 μg) were separated by SDS-PAGE and electrophoretically transferred to polyvinylidene difluoride membranes (Millipore, Bedford, MA). Then, the membranes were incubated with anti-AEP antibodies (R&D Systems Inc., Minneapolis, MN) at 4 °C overnight, followed by incubation with IRDye 680LT fluorescent secondary antibody (LI-COR Biosciences, Lincoln, NE). Proteins were visualized using the Odyssey Fc Imaging System (LI-COR Biosciences). Mouse β-actin was used as the loading control.

### Morris water maze test

After treatment, the SAMP8 and SAMR1 mice were trained in a round water pool (diameter 150 cm) with extra-maze cues. Each animal received four training trials per day for 5 consecutive days, to learn to find the hidden platform located 1.5 cm below the water surface. In each trial, the mice were given 60 s to find the platform in one of four different positions. The animals were allowed to stay at the platform for 10 s if they found the platform within the given time. However, if the animals failed to find the platform within the given time, they were manually guided to the platform and left there for 10 s. The escape latency was recorded for up to 60 s. After each trial, the mice were dried and kept in a warm cage. The inter-trial interval for each mouse was 10 min. For the probe test, the platform was removed and the mice were allowed to swim for 60 s. The probe test was conducted 24 h after the last training trial. Data were analyzed by an investigator who was blinded to the treatment condition.

### Immunofluorescent staining and quantification

Mice were deeply anesthetized and cardinally perfused with 4% paraformaldehyde (PFA) in phosphate-buffered saline (PBS). The entire brain was removed and cut into coronal sections at 10-μm thickness using a cryostat microtome (Leica CM1950, Germany) and stored at − 80 °C until staining. Immunofluorescence staining of phospho-tau, Iba1 and MAP-2 was performed as previously described [22]. Permeabilization of brain sections was performed in PBS with 0.1% Triton X-100 for 15 min at room temperature. After being blocked, the sections were incubated with primary antibody overnight at 4 °C. Next day, after three washes with PBS, the sections were incubated with the Alexa Fluor 488-conjugated donkey anti-rabbit or Alexa Fluor 594 anti-mouse IgG secondary antibody (Invitrogen Life Technologies, Carlsbad, CA). Photographs were taken and analyzed by using a Leica SP8 confocal microscope (Leica Microsystems, Germany). Quantification was carried out in six slices of each brain spaced 120 μm apart to estimate the average number of immune-labeled cells per unit area and the average intensity of the immunostaining. Quantification and analysis were conducted by an experimenter who was blinded to the treatment condition.

### Data analysis and statistical method

Data are expressed as mean ± SEM and were analyzed with the Prism 7 software (La Jolla, CA). The concentration of the inhibitor yielding half-maximal inhibition (IC_50_) of enzymatic activity was calculated using the following equation: Fractional Enzymatic Activity = Bottom + (Top-Bottom)/(1 + 10^((LogIC_50_-*C*)*n)), where *C* is the logarithm of inhibitor concentration and *n* is the Hill coefficient. The statistical difference between two independent groups was analyzed with the unpaired Student’s *t*-test, and that among more than two groups was assessed with the parametric one-way ANOVA followed by a Tukey’s *post-hoc* test. *Post-hoc* tests were conducted when the *F *value achieved the necessary level (*P* < 0.05) and there was no significant variance inhomogeneity. For the Morris water maze test, a two-way ANOVA repeated measures was used to compare the acquisition data. Differences were considered to be significant when *P* < 0.05.

## Results

### Expression and activity of AEP in the brains of SAMP8 and SAMR1 mice

**Fig. 1:**
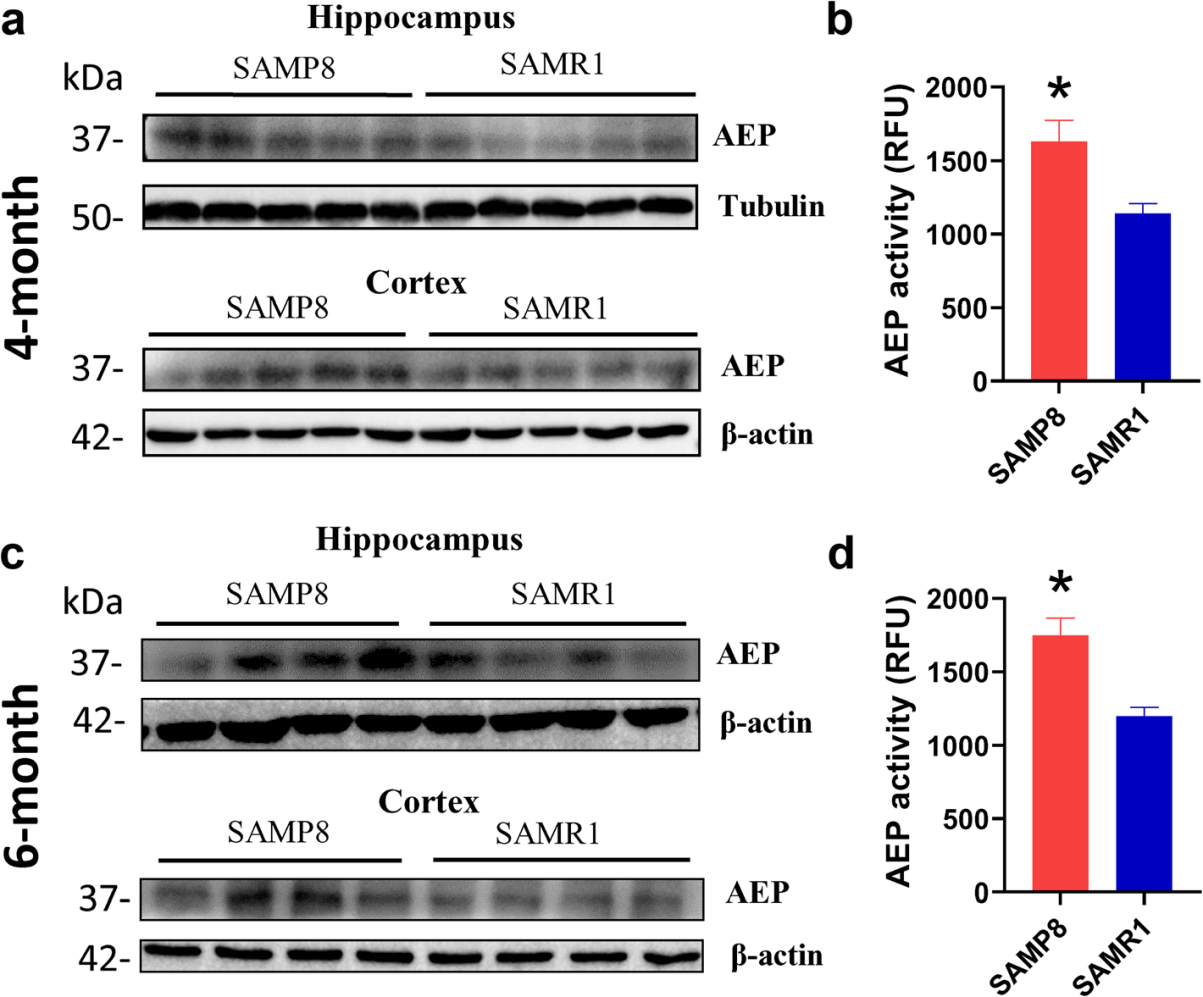
Protein expression and activity of AEP in brains of SAMP8 and SAMR1 mice. **a** Western blotting bands of mature AEP in the hippocampus and cortex of 4-month-old SAMP8 mice and age-matched SAMR1 mice. **b** Activity of AEP in the whole brains of 4-month-old SAMP8 and SAMR1 mice. * *P* < 0.05, Student’s *t*-test, *n* = 5 per group. **c** Mature AEP in the hippocampus and cortex of 6-month-old SAMP8 mice and age-matched SAMR1 mice. **d** AEP activity in the whole brains of 6-month-old SAMP8 and SAMR1 mice. * *P* < 0.05, unpaired Student’s *t*-test, *n* = 5 per group

We first assessed whether the mature/cleaved form of AEP differs between pathological and healthy conditions. It has been reported that both the expression and enzymatic activities of mature AEP in the brains of AD patients are higher than those in healthy individuals [8, 10, 12, 23]. Here we found that the active AEP fragments in the cortex and hippocampus of 4- and 6-month-old SAMP8 mice were elevated compared to the age-matched SAMR1 mice (Fig. 1a and c). The enzymatic activity of mature AEP in 4- and 6-month SAMP8 mice was significantly higher than that in age-matched SAMR1 mice (Fig. 1b and d).

### Inhibitory effect of δ-secretase inhibitor 11 on AEP activity *in vitro* and *in vivo*

**Fig. 2:**
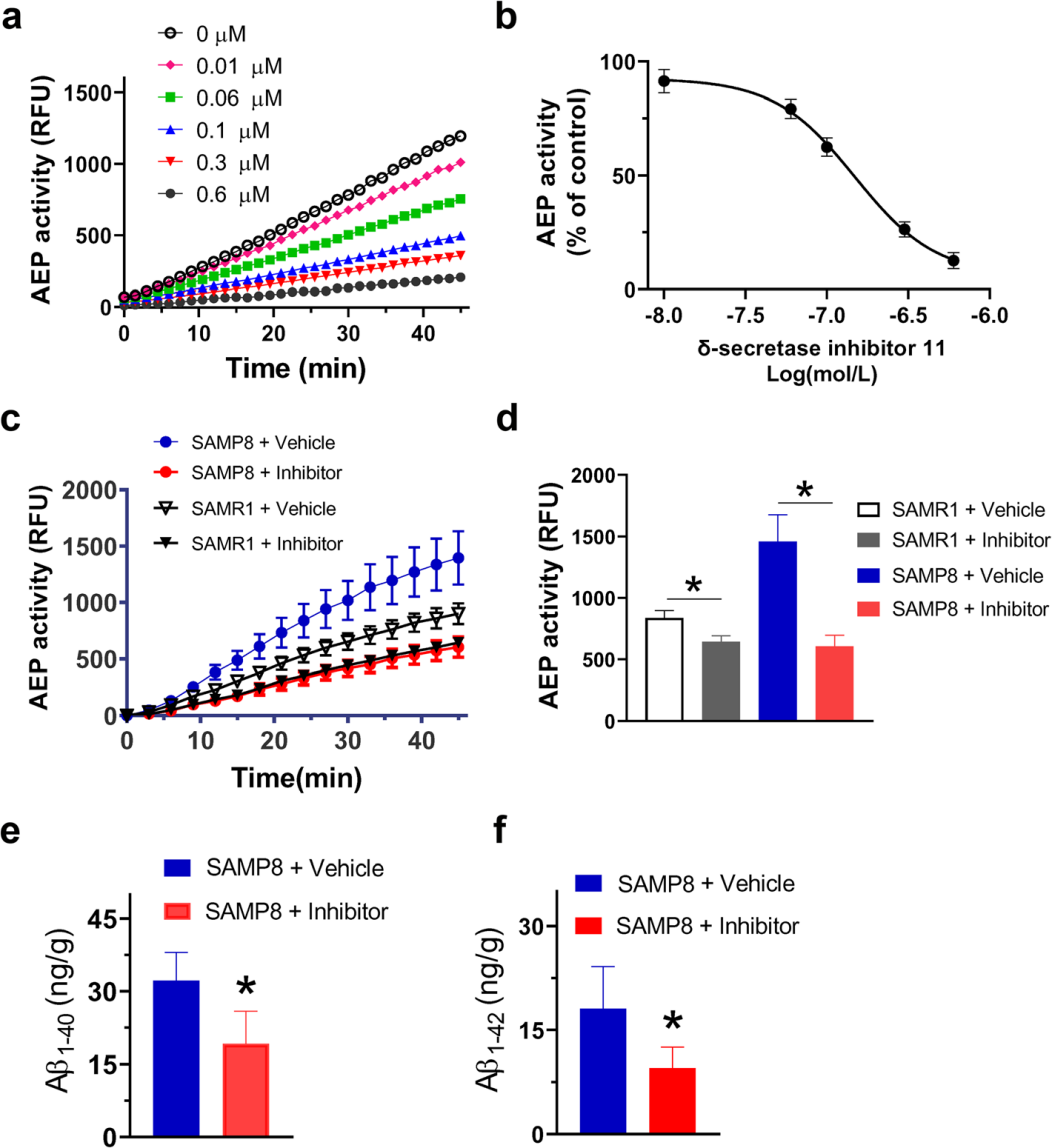
Pharmacological action of the δ-secretase inhibitor 11. **a** The enzymatic activity of AEP was measured by a 45-min fluorescent substrate cleavage assay. **b** The concentration-response of δ-secretase inhibitor 11 on AEP activity. Data were obtained at 45 min after the substrate cleavage reaction, *n* = 5 independent experiments. **c** The enzymatic activity of AEP in brains of SAMP8 and SAMR1 mice was measured by a 45-min fluorescent substrate cleavage assay. **d** AEP activity was obtained at 45 min after the substrate cleavage reaction and compared between groups. *P < 0.05, *n* = 6 mice per group, one-way ANOVA followed by a Tukey’s *post-hoc* test. **e**, **f** Measurement of Aβ_1–40_ and Aβ_1–42_ in the brains of SAMP8 mice treated with vehicle or δ-secretase inhibitor 11. *P < 0.05, unpaired Student’s *t*-test; *n* = 6 mice per group

The chemical structure of δ-secretase inhibitor 11 is illustrated in Figure S1. This δ-secretase inhibitor 11 has been reported to selectively inhibit AEP activity with an IC_50_ value of 0.70 ± 0.18 μM [14]; this effect is 46- to > 282-fold more potent than its inhibition over other cysteine proteases, including caspase-3, caspase-8, cathepsin-S and cathepsin-L [14]. Here, we tested again the inhibitory effect of δ-secretase inhibitor 11 on AEP activity *in vitro* and the dose-response relationship revealed that the δ-secretase inhibitor 11 had an IC_50_ value of 0.15 ± 0.09 μM (Fig. 2a and b).

To examine the chronic effect of δ-secretase inhibitor 11 on AEP activity in the brain, we treated 4-month-old SAMP8 and SAMR1 mice with δ-secretase inhibitor 11 (10 mg/kg) or vehicle once daily *via* oral gavage for 3 months. The body weight was recorded over the treatment period and did not show any difference among the four groups (Fig. S1). As the mammalian AEP is present in the brain, liver, kidney and many other tissues, AEP activity was measured in these tissues after the last treatment. We again found that AEP activity in the SAMP8 mouse brain was higher than that in the SAMR1 mouse brain. Oral administration of δ-secretase inhibitor 11 significantly suppressed brain AEP activity compared to the vehicle treatment (Fig. 2c and d). The concentrations of Aβ_1–40_ and Aβ_1–42_ in brain lysates of SAMP8 mice were significantly reduced due to AEP inhibition by δ-secretase inhibitor 11 (Fig. 2e and f). Interestingly, AEP activity (RFU ≈ 50,000) in kidney and liver tissues of these mice was nearly 20 times higher than that (RFU ≈ 2,500) in the brain tissue and was not altered by the 3-month treatment (Fig. S1).

### Systemic administration of δ-secretase inhibitor 11 prevented memory loss in SAMP8 mice

**Fig. 3:**
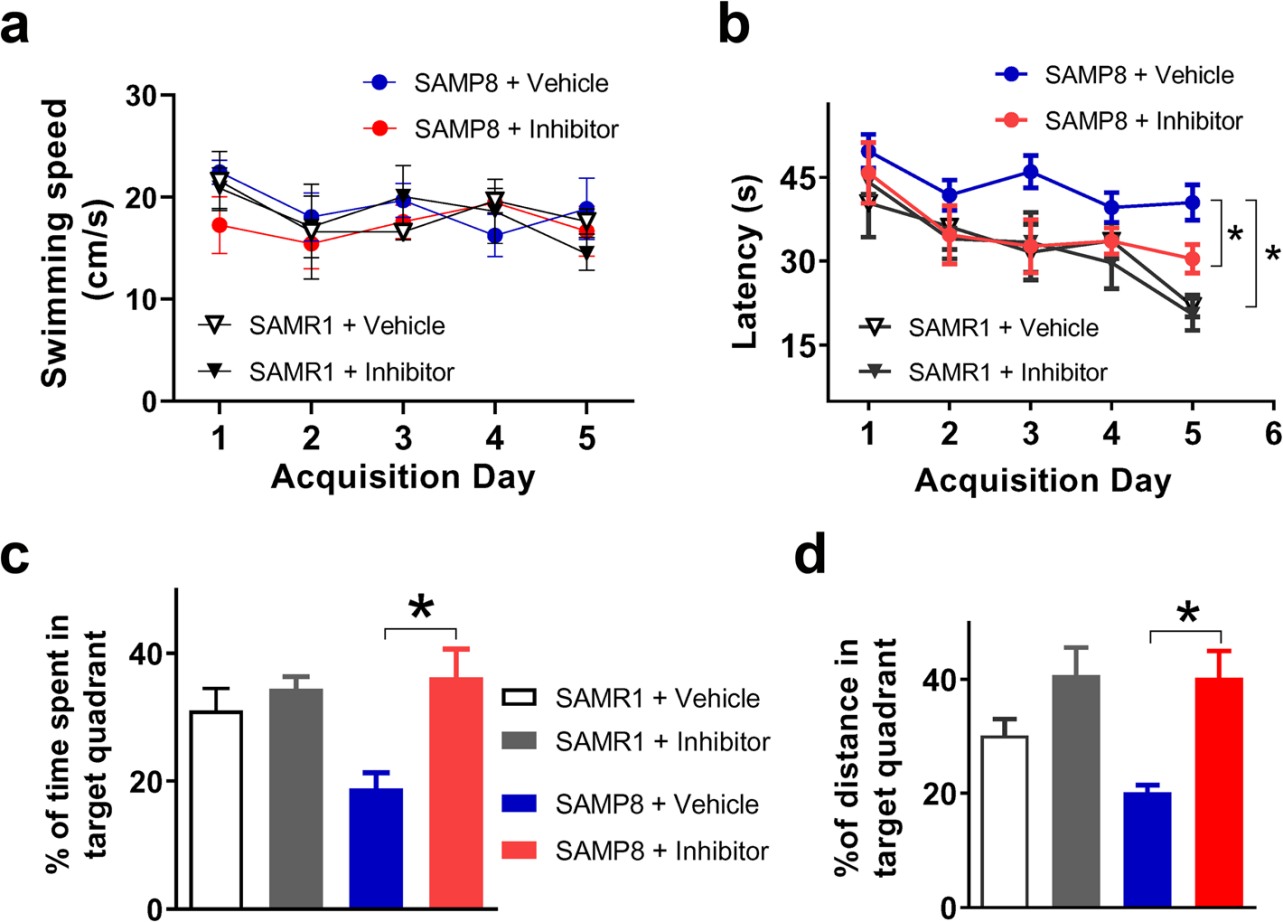
Effect of δ-secretase inhibitor 11 treatment on memory function of mice. **a**, **b** The swimming speed and escape latency of mice over the 5-day acquisition training. Two-way ANOVA analysis revealed the time effect (*F* (2.922, 35.06) = 9.659, *P* < 0.001) and group effect (*F* (3, 12) = 7.355, *P* < 0.05) on escape latency. SAMP8 mice learned significantly slower than SAMR1 mice (*P* < 0.05), and the vehicle-treated SAMP8 mice learned significantly slower than the δ-secretase inhibitor 11-treated SAMP8 mice (*P* < 0.05). *n* = 9 mice per group. **c**, **d** The percentage of time spent and the distance travelled in the target quadrant in the probe test, which was performed on day 6. *P < 0.05, *n* = 9 mice per group, one-way ANOVA followed by a Tukey’s *post-hoc* test

Given that the systemic administration of δ-secretase inhibitor 11 efficiently repressed brain AEP activity, we examined whether this reagent had therapeutic efficacy in this SAD model. We treated 4-month-old SAMP8 and SAMR1 mice with δ-secretase inhibitor 11 (10 mg/kg, p.o.) once daily *via* oral gavage for 3 months, and then conducted Morris water maze test to assess cognitive function related with spatial learning and memory [24]. Results showed that the swimming speed did not differ among animals treated with either vehicle or δ-secretase inhibitor 11 (Fig. 3a). The escape latency of each group decreased progressively over the 5-day training period (Fig. 3b), suggesting the development of spatial learning and memory. The SAMP8 mice learned slower than the SAMR1 mice (*P* < 0.05), while the SAMP8 mice treated with δ-secretase inhibitor 11 learned significantly faster than the vehicle-treated SAMP8 mice (*P* < 0.05). In the probe test on day 6, the SAMP8 mice treated with δ-secretase inhibitor 11 spent significantly longer time and travelled a longer distance in the target quadrant than the vehicle-treated SAMP8 mice (Fig. 3c, d). These data indicated that the 3-month treatment with δ-secretase inhibitor 11 ameliorated the cognitive impairment of SAMP8 mice.

### Treatment with δ-secretase inhibitor 11 attenuated AD-like pathologies

**Fig. 4:**
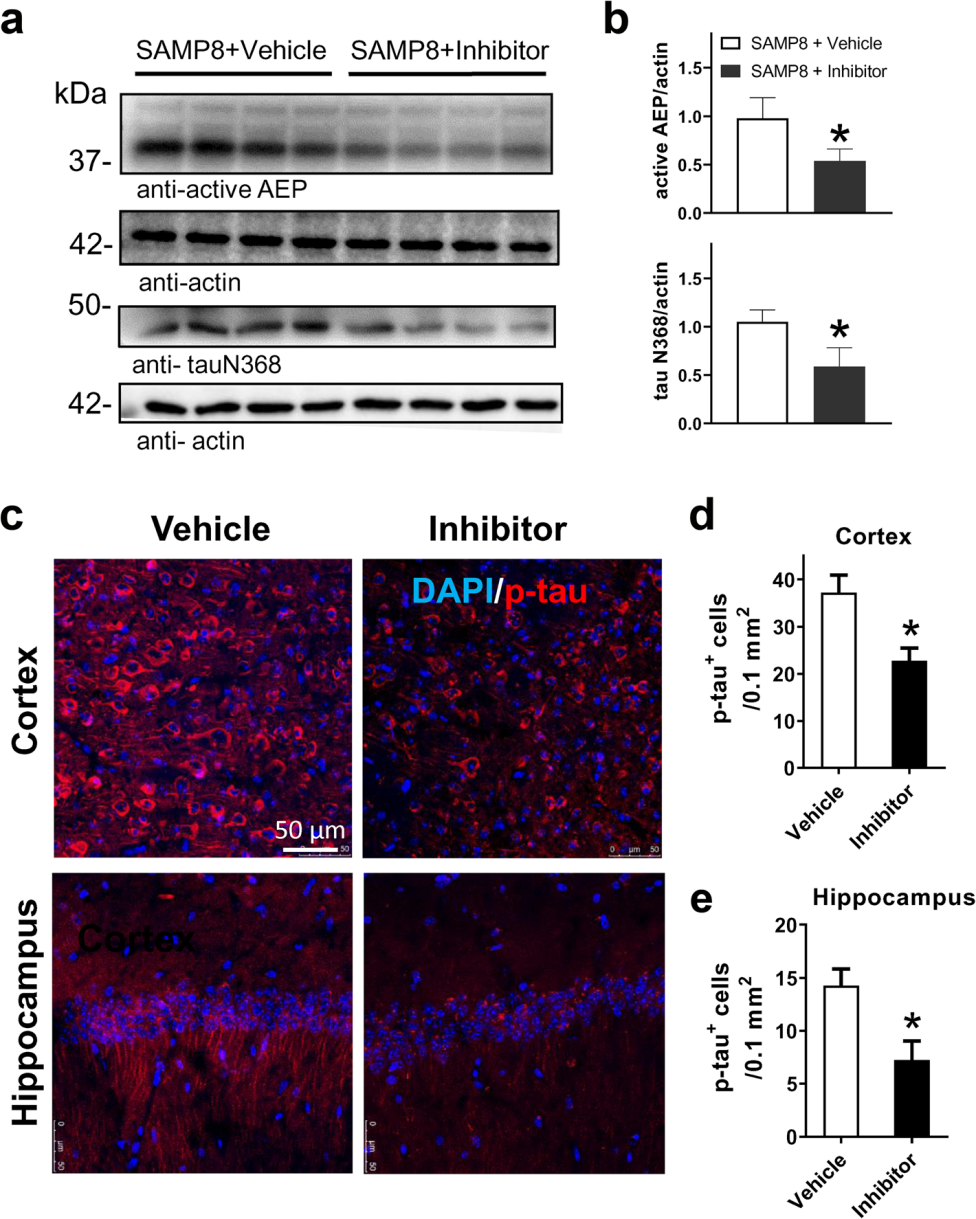
Treatment with δ-secretase inhibitor 11 reduced pathological changes of tau in SAMP8 mice. **a**, **b** Western blot analysis of AEP and tau N368. *P < 0.05, unpaired Student’s *t*-test; *n* = 4 mice per group. **c** Immunostaining of phospho-tau (Thr231) in cortical and hippocampal neurons. **d**, **e** Density of phospho-tau-positive cells in the cortex and hippocampus of SAMP8 mice. *P < 0.05 between groups, unpaired Student’s *t*-test; *n* = 5 mice per group

We next investigated the cellular mechanisms involved in the therapeutic efficacy of δ-secretase inhibitor 11 in SAMP8 mice. Previous studies have revealed that the activated AEP induces hyperphosphorylation of tau by cleaving tau protein at N255 and N368, and the tau N368 fragments have been detected in human AD brains [12, 13]. Western blot analysis showed that the protein levels of AEP and tau N368 were significantly suppressed after 3-month treatment with δ-secretase inhibitor 11 (Fig. 4a, b). Immunofluorescence staining showed that the phospho-tau was significantly attenuated in the cortex and hippocampus of SAMP8 mice with δ-secretase inhibitor 11 treatment (Fig. 4c-e).

**Fig. 5:**
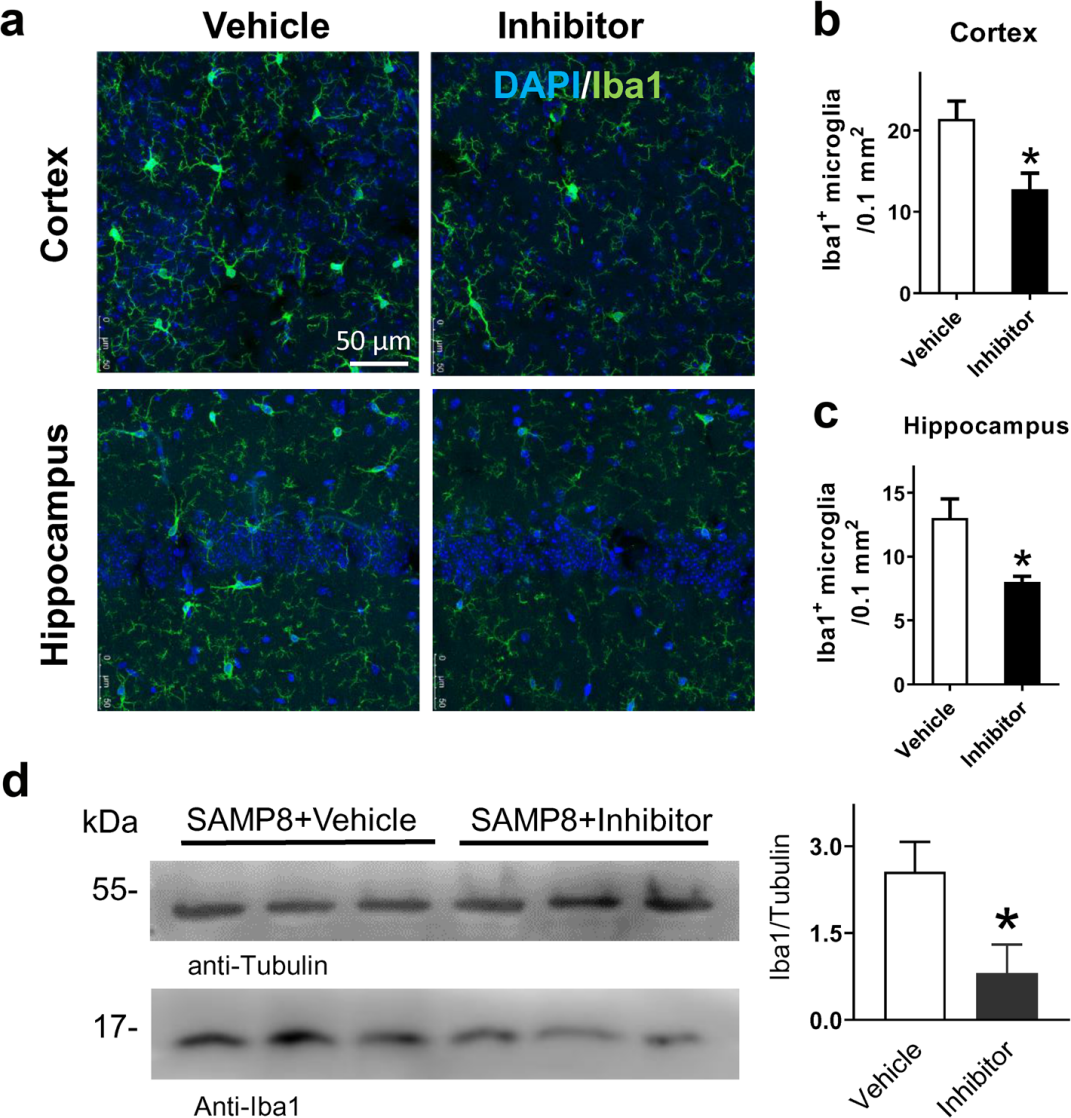
Treatment with δ-secretase inhibitor 11 reduced pathological activation of microglia in SAMP8 mice. **a** Immunostaining of Iba1, a marker of microglial activation, in the cortex and hippocampus. **b**, **c** Density of Iba1-positive cells in the cortex and hippocampus of SAMP8 mice. *P < 0.05, between groups. *n* = 5 mice per group, unpaired Student’s *t*-test. **d** Western blot analysis of Iba1. *P < 0.05, unpaired Student’s *t*-test; *n* = 6 mice per group

**Fig. 6:**
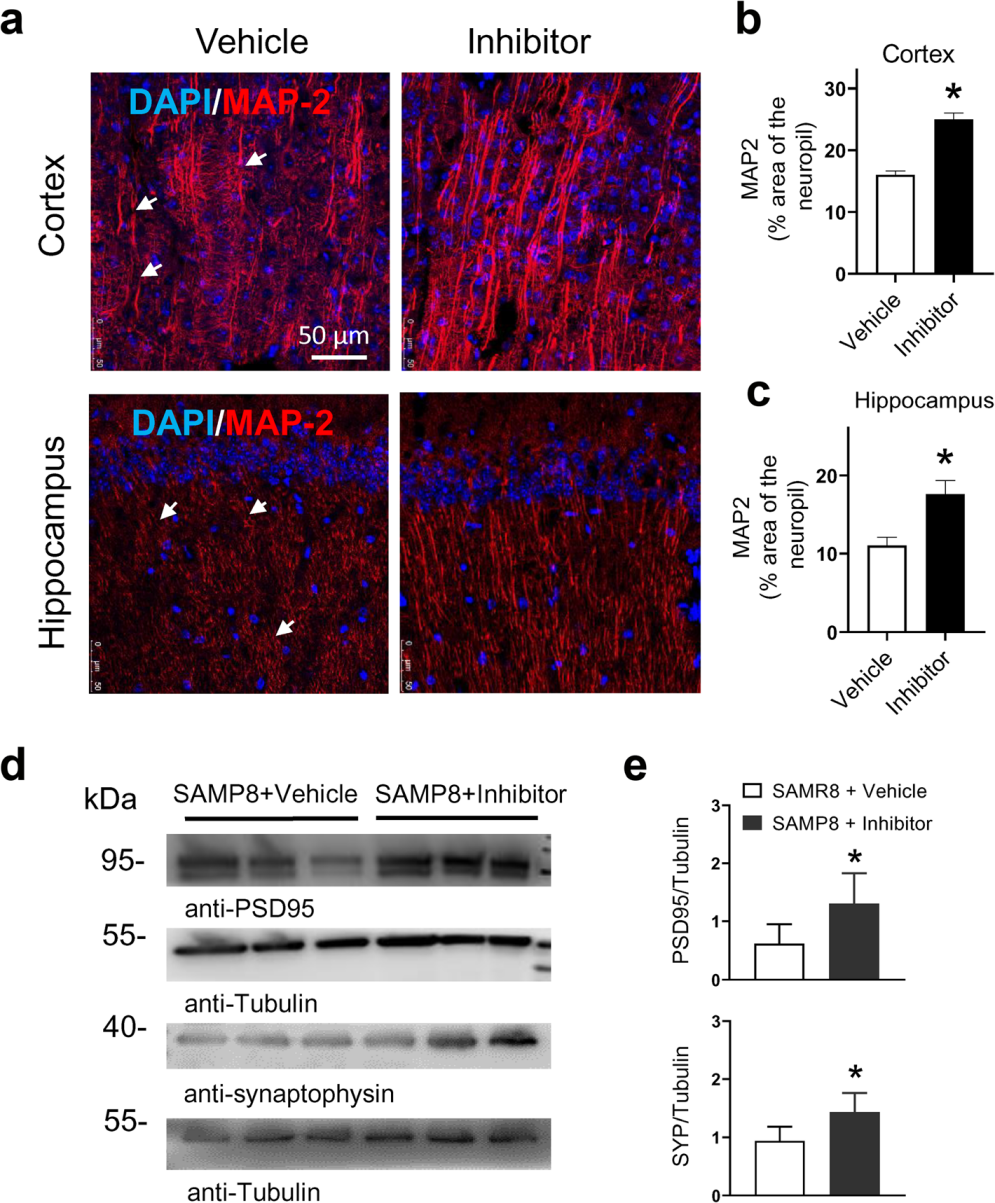
Treatment with δ-secretase inhibitor 11 restored MAP-2 and synapse-associated protein expression in SAMP8 mice. **a** Immunostaining of the neuronal marker MAP-2 in the cortex and hippocampus. Arrows indicate neuronal soma and dendrites stained by MAP-2. **b**, **c** MAP-2 immunoreactivity was illustrated as the percent area of the neuropil in the cortex and hippocampus of SAMP8 mice. *P < 0.05, unpaired Student’s *t*-test, *n* = 5 mice per group. **d**, **e** Western blot analysis of brain lysate showing the expression of postsynaptic density protein 95 (PSD-95) and synaptophysin (SYP) in the two groups. *P < 0.05 between groups, unpaired Student’s *t*-test; *n* = 6 mice per group

Tau hyperphosphorylation has been reported to drive microglial activation in the cortex of AD mice [25]. Therefore, we assessed microglial activation in SAMP8 mouse brain using Iba1 staining. We found that the Iba1 signal was markedly decreased in the cortex and hippocampus of SAMP8 mice treated with δ-secretase inhibitor 11 (Fig. 5a-c). Whole-brain lysate analysis showed that the level of Iba1 was significantly reduced by δ-secretase inhibitor 11 (Fig. 5d). To detect pathological changes of neuronal structure in SAMP8 mouse brain, we performed immunofluorescence staining of neuronal marker MAP-2, which mainly localizes in the soma and dendrites of most neurons. MAP-2 immunoreactivity was expressed in a fragment pattern in the cortex and hippocampus of vehicle-treated mice, and the MAP-2 density was significantly lower than that in SAMP8 mice treated with δ-secretase inhibitor 11 (Fig. 6a-c). Whole-brain lysate analysis showed that both synapse-associated protein SYP and PSD-95 were significantly up-regulated by drug treatment (Fig. 6d, e). These data indicated that the dendritic disruption and synaptic loss in SAMP8 mouse brain were substantially alleviated by treatment with δ-secretase inhibitor 11.

## Discussion

Over the past 20 years, many efforts have been made to modify tau and Aβ pathologies of AD by reducing the cytotoxicity of tau, preventing the aggregation of Aβ, and enhancing the clearance of Aβ, etc. [1, 4]. However, these attempts have all failed in phase 2 or 3 of clinical trials [26]. A possible reason is that the previous agents were tested to interfere with very late stage of AD disease [4].

The lysosomal AEP has been reported to mediate the AD pathogenesis through triggering tau and APP cleavage in an age-dependent manner [10–12]. It has been confirmed that the enzymatic activity of AEP in the brains of AD patients is higher than that in healthy persons of the same age [10, 12, 23]. Here we showed that the expression and enzymatic activity of mature AEP were increased in the brains of SAMP8 mice compared to the age-matched SAMR1 mice. Based on the previous reports that inhibition of AEP is a new strategy that interferes with the early stage of AD disease [7, 9, 27], we applied a selective AEP inhibitor δ-secretase inhibitor 11 to 4-month-old SAMP8 mice to test whether it could modify the brain pathology related to aging and AD. In addition, the δ-secretase inhibitor 11 is a selective and potent AEP inhibitor that can penetrate the BBB of mice.

We found that 3-month systemic administration of δ-secretase inhibitor 11 markedly suppressed AEP activation in the brains of SAMP8 mice and attenuated memory loss, while having no effect on body weights of these mice, suggesting a low toxic nature of this reagent. The amelioration of cognitive decline by δ-secretase inhibitor 11 implies that the inhibition of AEP could inhibit the AD-like disease progression. Indeed, brain lysate analysis and morphological characterization demonstrated that the δ-secretase inhibitor 11 reduced the production of Aβ_1–40/42_, prevented the pathological changes of tau protein and suppressed microglial activation, a hallmark of neuroinflammation [28]. Moreover, the δ-secretase inhibitor 11 prevented synaptic loss and restored the expression of MAP-2, which is a dendritic protein and an indicator of synaptic plasticity. This improved structural synaptic plasticity may underlie the amelioration of cognitive decline in SAMP8 mice.

A set of RNA sequencing data distributed by the National Center for Biotechnology Information shows that the gene expression of AEP (gene name: *LGMN*) is abundant in human tissues such as placenta, kidney, spleen, liver, thyroid, and gall bladder, but very low in normal brain tissue (https://www.ncbi.nlm.nih.gov/gene/5641). Similarly, the enzymatic activity of AEP in brain tissues of SMAP8 or SAMR1 mice is almost 20 times lower than that in the kidney and liver (Fig. S1). The above information strongly implies that the upregulation of AEP activity in a certain brain region can be considered as an abnormal condition and can be targeted for the intervention of neurodegeneration, such as the AD pathogenesis.

## Conclusion

The present preclinical study provided evidence that the up-regulated AEP in the brain is a potential and promising target for early treatment of AD. Inhibition of brain AEP would not incur severe side effects because AEP activity is much higher in peripheral organs. Systemic administration of the δ-secretase inhibitor 11 was effective in mitigating AD-like neurodegeneration but did not alter the AEP activity in liver and kidney. To promote translation of this new anti-AD strategy into a real therapy, AEP inhibitors with better pharmacokinetic properties than δ-secretase inhibitor 11 are waiting to be developed. Particularly, the plasma elimination half-life (t_1/2_) of δ-secretase inhibitor 11 in mice is about 2.31 h after an oral administration [14]. Ideally drugs with a longer half-life are desirable for diseases of the central nervous system, so that dosing can be maintained at a consistent level. For example, memantine has a plasma t_1/2_ of 60–80 h and is being used to treat patients with moderate-to-severe AD [29, 30]. New AEP inhibitors are required to have increased oral bioavailability, prolonged plasma t_1/2_, and enhanced brain-to-plasma distribution ratio.

## Supplementary Information

**Additional file 1: Figure S1.** Effect of δ-secretase inhibitor 11 on body weight and AEP activity.

## Data Availability

The datasets used and/or analysed in this study are available from the corresponding author on reasonable request.

## Abbreviations

AD: Alzheimer’s disease
SAD: Sporadic AD
FAD: Familial AD
AEP: Asparaginyl endopeptidase
Aβ: Amyloid β-protein
APP: Amyloid precursor protein
BBB: Blood-brain barrier
MAP-2: Microtubule-associated protein 2
PSD-95: Postsynaptic density protein 95
SYP: Synaptophysin
