# Supplementary material for: Pharmacological inhibition of asparaginyl endopeptidase by δ-secretase inhibitor 11 mitigates Alzheimer’s disease-related pathologies in a senescence-accelerated mouse model
Source: Transl Neurodegener. 2021 Mar 31;10:12. doi: 10.1186/s40035-021-00235-4 (PMC8015189; doi:10.1186/s40035-021-00235-4)
Supplement: Supplementary file 1 — Additional file 1: Figure S1. Effect of δ-secretase inhibitor 11 on body weight and AEP activity. [file 40035_2021_235_MOESM1_ESM.pdf]

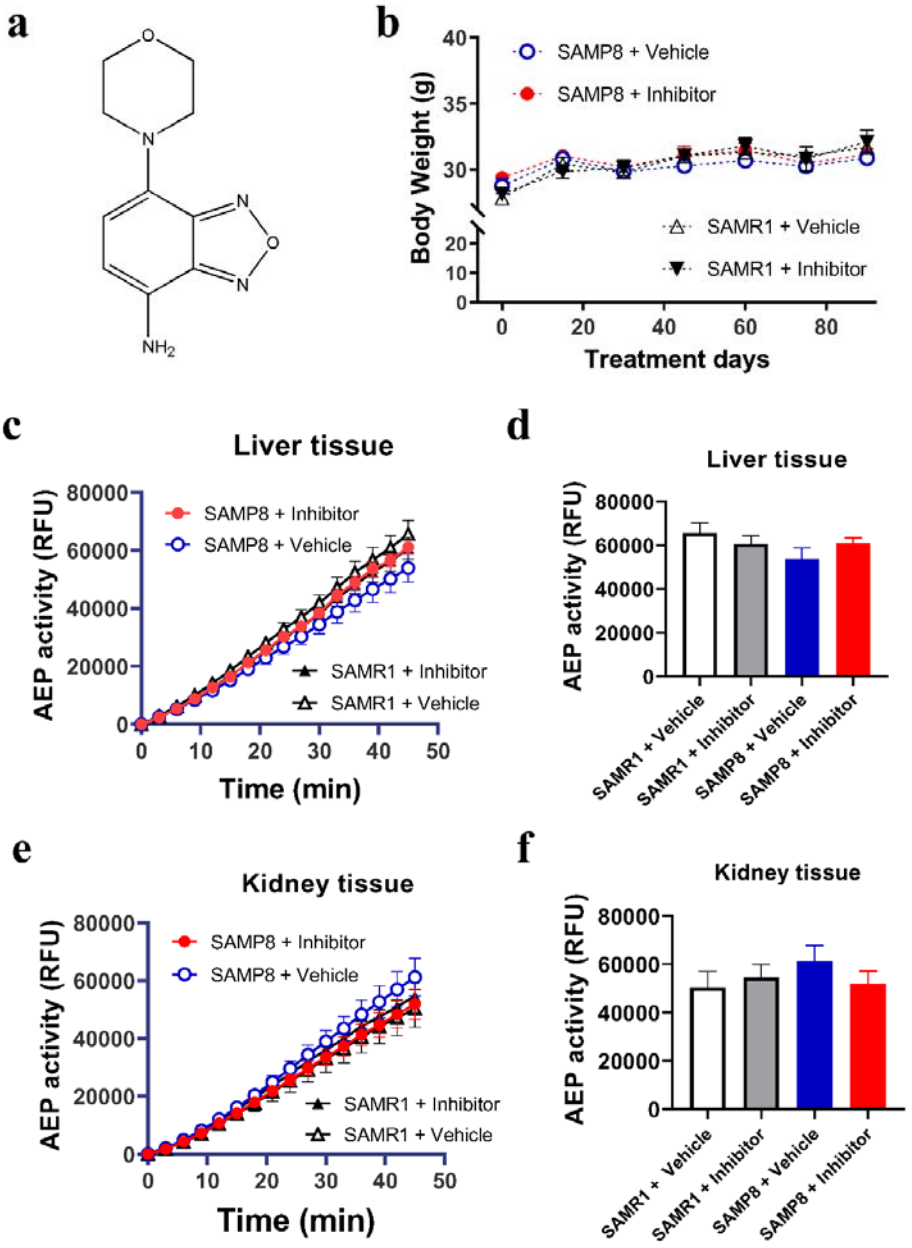

Fig. S1. Effect of δ-secretase inhibitor 11 on body weight and AEP activity. **a** Chemical structure of δ-secretase inhibitor 11. **b** Body weight records of SAMP8 and SAMR1 mice over the treatment period. $n = 9$ mice per group. **c** The enzymatic activity of AEP in the liver tissues of SAMP8 and SAMR1 mice was measured for 45 min. Mice had been treated with δ-secretase inhibitor 11 (10 mg/kg) for 3 months. **d** AEP activity was obtained at 45 min after the substrate cleavage reaction. $n = 6$ mice per group. **e, f** Measurement of AEP activity in

kidney tissues of SAMP8 and SAMR1 mice. $n = 6$ mice per group.
